# LCZ696 ameliorates lipopolysaccharide-induced endothelial injury

**DOI:** 10.18632/aging.202692

**Published:** 2021-04-11

**Authors:** Aihong Gao, Yu Wang, Xiao Gao, Wei Tian

**Affiliations:** 1 Department of Cardiology, NO.215 Hospital of Shaanxi Nuclear Industry, Xianyang 712000, China; 2 Department of Pathology, NO.215 Hospital of Shaanxi Nuclear Industry, Xianyang 712000, China

**Keywords:** lipopolysaccharide (LPS), LCZ696, endothelial cells, NF-κB, inflammation

## Abstract

Lipopolysaccharide (LPS)-induced endothelial dysfunction plays an important role in the pathogenesis of cardiovascular diseases. LCZ696, the dual-acting angiotensin receptor blocker, and neprilysin inhibitor has been used for the treatment of heart failure with reduced ejection fraction. Recent work suggests that LCZ696 therapy might have an anti-inflammatory effect in cardiovascular tissue. In the current study, we show that LCZ696 attenuates LPS-induced oxidative stress by reducing the production of intracellular reactive oxygen species (ROS) and the measurements of malonyl dialdehyde (MDA) level in human umbilical vascular endothelial cells (HUVECs). LCZ696 inhibits LPS-induced expressions and secretions of the pro-inflammatory cytokines, interleukin-6 (IL-6), interleukin-1α (IL-1α), and tumor necrosis factor β (TNF-β) as well as the chemokines, monocyte chemotactic protein 1 (MCP-1), and chemokine (C-X-C motif) ligand 1 protein (CXCL1). Additionally, we found that LCZ696 reduces LPS-induced expressions of vascular cell adhesion molecule 1 (VCAM-1) and P-selectin and the attachment of U937 monocytes to HUVECs. Mechanistically, LCZ696 prevents LPS-induced activation of the TLR4/Myd88 pathway and nuclear translocation of nuclear factor kappa-B (NF-κB) p65 factor. Based on these findings, we conclude that LCZ696 is capable of ameliorating LPS-induced endothelial dysfunction via anti-inflammatory properties.

## INTRODUCTION

Inflammation is one of the key underlying mechanisms for various human diseases viewed as the body’s response to invading pathogens or endogenous signals, which could promote tissue repair or sometimes have detrimental consequences. In cardiovascular tissue, inflammation plays a critical role in the pathological process of atherosclerosis and other cardiovascular diseases. Vascular inflammation is involved in all stages of disease development, starting from the immune cell attachment to the vascular wall to the advanced atheroma formation [1]. Endothelial cells are important players in inflammatory reactions. While quiescent endothelial cells act to inhibit inflammation, activated endothelial cells often switch their original phenotype to promote inflammation. Vascular inflammation causes high production of reactive oxygen species (ROS) in the vessel wall, which is a direct insult to the vessel wall, and the inflammation associated with ROS is a critical contributor to endothelial dysfunction [2]. Endothelial cell dysfunction is characterized by abnormal vascular dilation, high coagulation status, and increased vascular permeability. These pathological processes are mediated by increased production of pro-inflammatory cytokines and chemokines and increased leukocytes activation and adhesion to the vascular wall [3]. Lipopolysaccharide (LPS), also known as endotoxin, is the major component of the cell membrane in Gram-negative bacteria. LPS can activate endothelial cells and induce the expression of inflammatory mediators [4]. LPS activates endothelial cells by binding to Toll-like receptor 4 complex (TLR4) and recruits the adaptor protein myeloid differentiation factor (MyD88) to mediate MyD88-dependent pathways which ultimately activate the nuclear factor-kappa B (NF-κB) and endothelial injury [5].

Natriuretic peptide (NP) is the substance released from heart tissue. The high level of the NP system is an important indicator of heart diseases. One of the key components of the NP system is neprilysin, which catalyzes the degradation of NP proteins and Angiotensin II. Neprilysin inhibitors have been useful in the treatment of chronic heart failure [6]. Angiotensin receptor blocker (ARB) types of drugs have been used for the treatment of hypertension and congestive heart failure for many years. A recent clinical trial indicates that the combination of ARB and neprilysin inhibition have a better outcome than ARB alone, in the treatment of heart failure [7]. LCZ696 is a newly approved first-in-class drug that combines angiotensin receptor blockade and neprilysin inhibition to treat heart failure with reduced ejection fraction (HFrEF) [8]. LCZ696 is a combination of sacubutril and valsartan. Valsartan blocks Angiotensin II receptor AT1, which causes blood vessel dilation. Sacubitril is the prodrug which can be activated to inhibit neprilysin. The dual effect of LCZ696 causes blood vessel dilation and reduction of the expansion of extracellular fluid volume, which improves heart function in HFrEF patients [9]. LCZ696 has been shown to possess anti-inflammation and anti-oxidative stress effects in cardiovascular disease models *in vivo* and *in vitro* [10, 11]. In endothelial cells, AT1-Receptor inhibition possesses a beneficial effect on improving endothelial function [12, 13]. The administration of LCZ696 shows improved endothelial dysfunction in hypertensive animals [14]. These facts suggest that the dual inhibition of AT1 and neprilysin exerts a beneficial effect in vascular endothelial cells. In this study, we investigated the underlying protective mechanism of LCZ696 in endothelial cells.

## RESULTS

### Cytotoxicity of LCZ696

**Figure 1:**
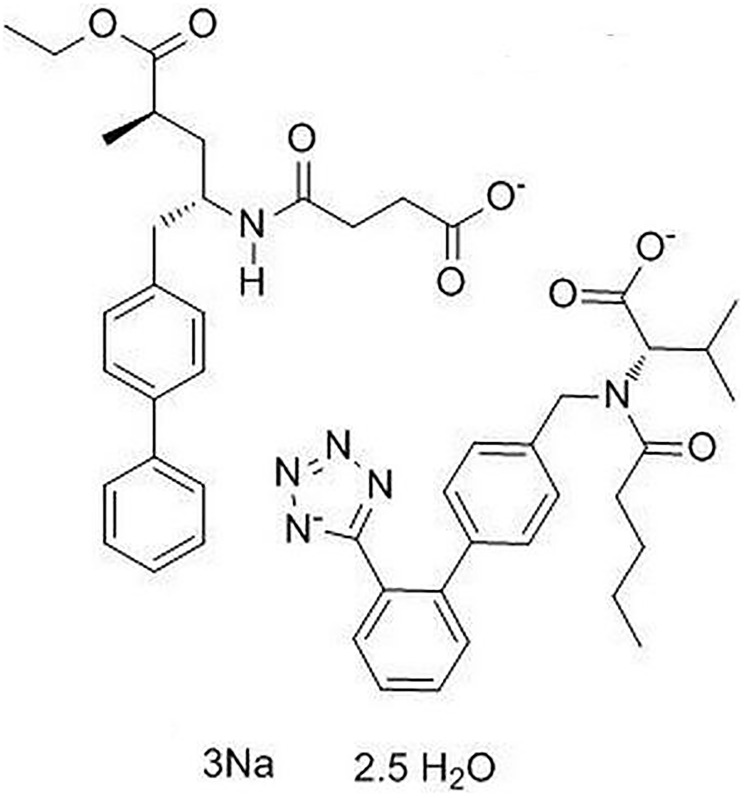
Molecular structure of LCZ696.

**Figure 2:**
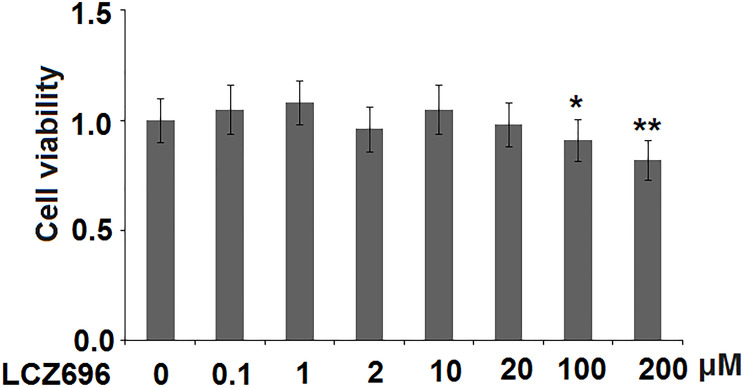
The effects of LCZ696 on cell viability in human umbilical vein endothelial cells (HUVECs). HUVECs were stimulated with LCZ696 at concentrations of 0.1, 1, 2, 10, 20, 100, and 200 μM for 24 hours. Cell viability was measured using MTT assay (^*, **^P < 0.05, 0.01 vs. control group).

LCZ696 is a co-crystallized complex with a combination of sacubitril and valsartan, in a one-to-one molar ratio, one LCZ696 molecule consists of six sacubitril anions, six valsartan anions, 18 sodium cations, and 15 molecules of water. The molecular structure of LCZ696 is shown in Figure 1. Valsartan is an angiotensin II receptor blocker and sacubitril is activated to be a neprilysin inhibitor. To determine the cytotoxicity of LCZ696, HUVECs were treated with a series of doses of LCZ696 ranging from 0.1 to 200 μM for 24 hours. Results show that when the concentration of LCZ696 was lower than 100 μM, it did not affect the cell viability of HUVECs. However, when the concentration of LCZ696 was at 100 and 200 μM, it significantly reduced the cell viability by 9% and 18%, respectively (Figure 2). Based on these observations, we treated HUVECs with 10 and 20 μM LCZ696 in the subsequent experiments.

### LCZ696 mitigates LPS-induced oxidative stress

**Figure 3:**
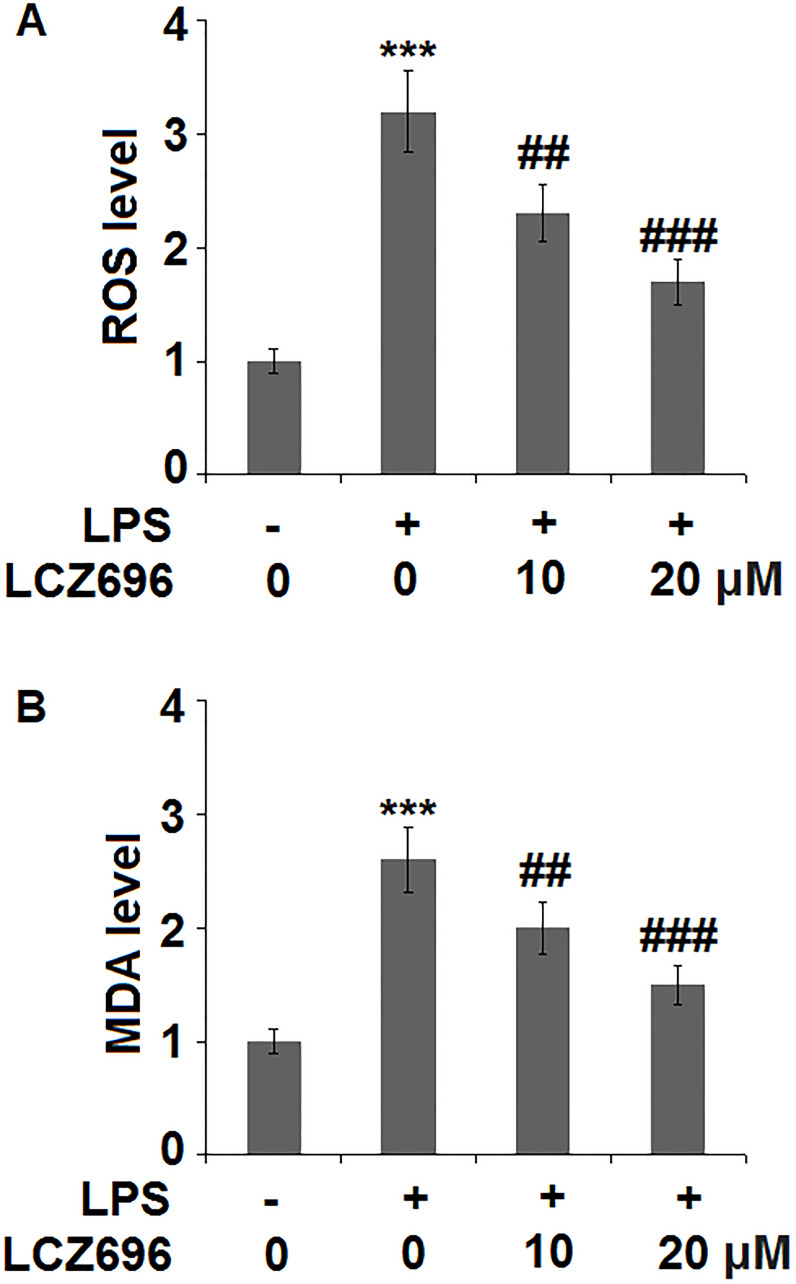
LCZ696 mitigates lipopolysaccharide (LPS)-induced oxidative stress in HUVECs. Cells were stimulated with LPS in the presence or absence of LCZ696 (10, 20 μM) for 24 hours. (**A**). ROS) was measured using DCFH-DA staining; (**B**). The level of MDA (^***^P < 0.005 vs. control group; ^##, ###^*P* < 0.01, 0.005 vs. LPS group).

Subsequently, the effect of LCZ696 in oxidative stress in endothelial cells was examined. As shown in Figure 3A, LPS treatment alone induced about 3.2-fold high of ROS, while the addition of 10 and 20 μM of LCZ696 suppressed LPS-induced ROS production to 2.3- and 1.7-fold, respectively. Meanwhile, our data shows that LCZ696 had a similar suppressive effect on another oxidative marker-MDA. As shown in Figure 3B, LPS induced about 2.6-fold high MDA activity, while the presence of two doses of LCZ696 reduced its activity only to 2- and 1.5-fold, respectively.

### LCZ696 inhibits LPS-induced cytokine production

**Figure 4:**
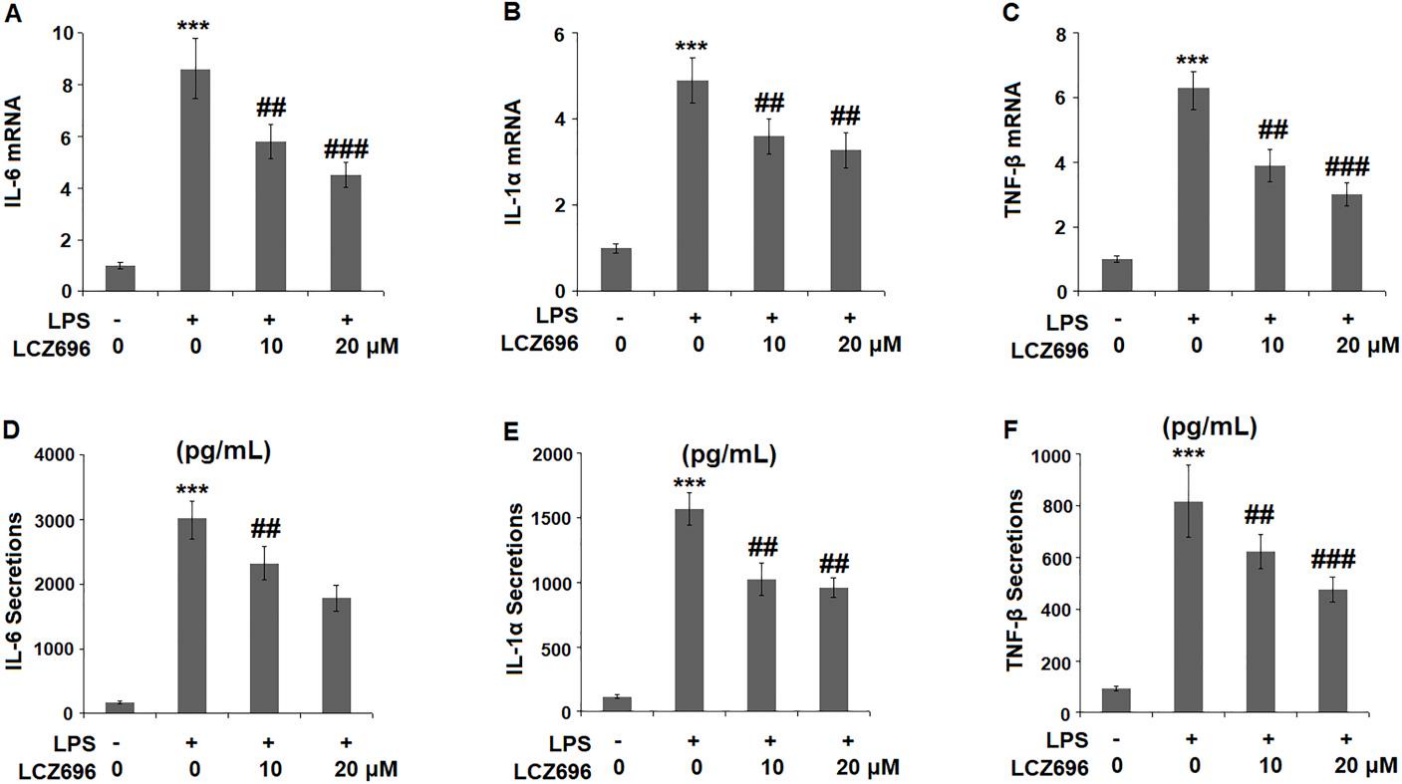
LCZ696 inhibits LPS-induced expression and production of pro-inflammatory cytokines in HUVECs. Cells were stimulated with LPS in the presence or absence of LCZ696 (10, 20 μM) for 24 hours. (**A**). mRNA of IL-6; (**B**). mRNA of IL-1α; (**C**). mRNA of TNF-β; (**D**) Secretions of IL-6; (**E**). Secretions of IL-1α; (**F**). Secretions of TNF-β (^***^P < 0.005 vs. control group; ^##, ###^*P* < 0.01, 0.005 vs. LPS group).

To evaluate the consequence of LCZ696 on LPS-induced inflammation, the expression profiles of several key pro-inflammatory cytokines were examined. At mRNA level, LPS alone induced elevated transcription of IL-6, IL-1α, and TNF-β, however, the presence of two doses of LCZ696 showed notable suppression on the transcription of these cytokine genes (Figure 4A–4C). Through ELISA assay, the suppression by LCZ696 of the production of these cytokines was confirmed (Figure 4D–4F). LPS treatment stimulated the secretions of IL-6, IL-1α, and TNF-β, causing an increase from 166.8, 115.3, and 93.1 pg/ml to 3025.6, 1568.1, and 817.9 pg/ml, respectively. However, 10 μM LCZ696 reduced the secretions of these pro-inflammatory cytokines to 2321.7, 1025.6, and 623.5 pg/ml, which were further decreased to 1786.5, 735.5, and 476.2 pg/ml by 20 μM LCZ696, respectively.

### LCZ696 inhibits LPS-induced chemokine production

**Figure 5:**
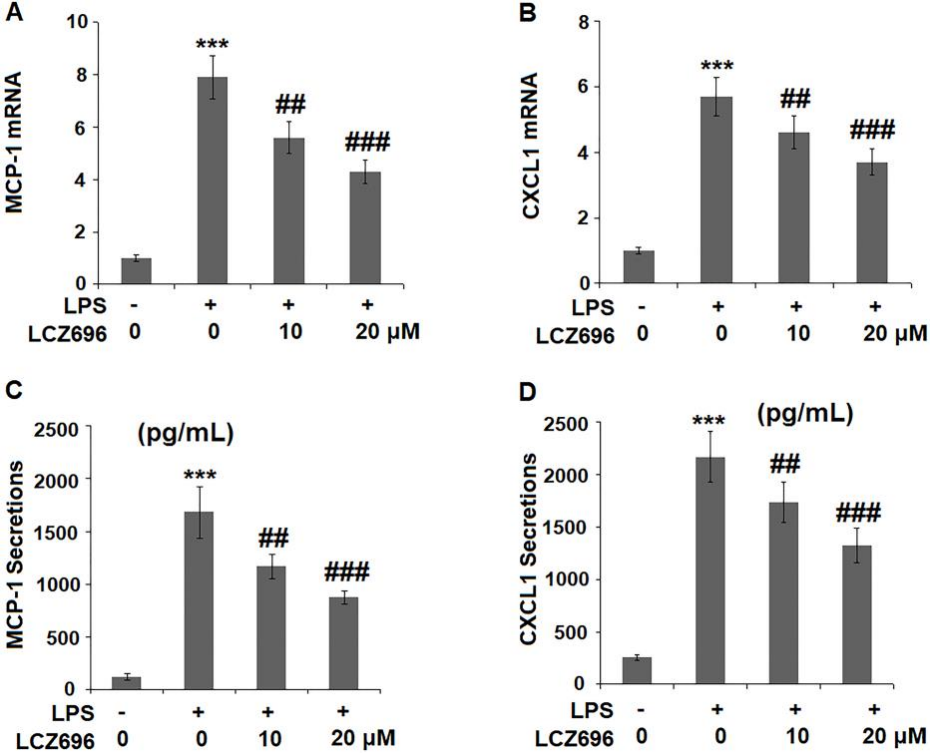
LCZ696 reduced LPS-induced expression and production of chemokines MCP-1 and CXCL1 in HUVECs. Cells were stimulated with LPS (1 μg/mL) in the presence or absence of LCZ696 (10, 20 μM) for 24 hours. (**A**). mRNA of MCP-1; (**B**). mRNA of CXCL1; (**C**). Secretions of MCP-1; (**D**). Secretions of CXCL1 (^***^P < 0.005 vs. control group; ^##, ###^*P* < 0.01, 0.005 vs. LPS group).

The expression levels of two primary chemokines CXCL1 and MCP-1 were also examined. At mRNA level, LPS alone caused a 7.9- and 5.7-fold high of MCP-1 and CXCL1. However, higher doses of LCZ696 reduced their induction to only 4.3- and 3.7-fold, respectively (Figure 5A, 5B). Similarly, LPS treatment increased the protein levels of MCP-1 and CXCL1 from 123.5 and 256.8 pg/ml to 1683.6 and 2167.3 pg/ml, which were reduced to 1173.4 and 1735.8 pg/ml by 10 μM LCZ696, and to 875.2 and 1325.8 pg/ml by 20 μM LCZ696, respectively. Thus, LCZ696 exerted a strong inhibitory effect against LPS-induced expression of pro-inflammatory chemokines.

### LCZ696 inhibits LPS-induced expression of cell adhesion molecules

**Figure 6:**
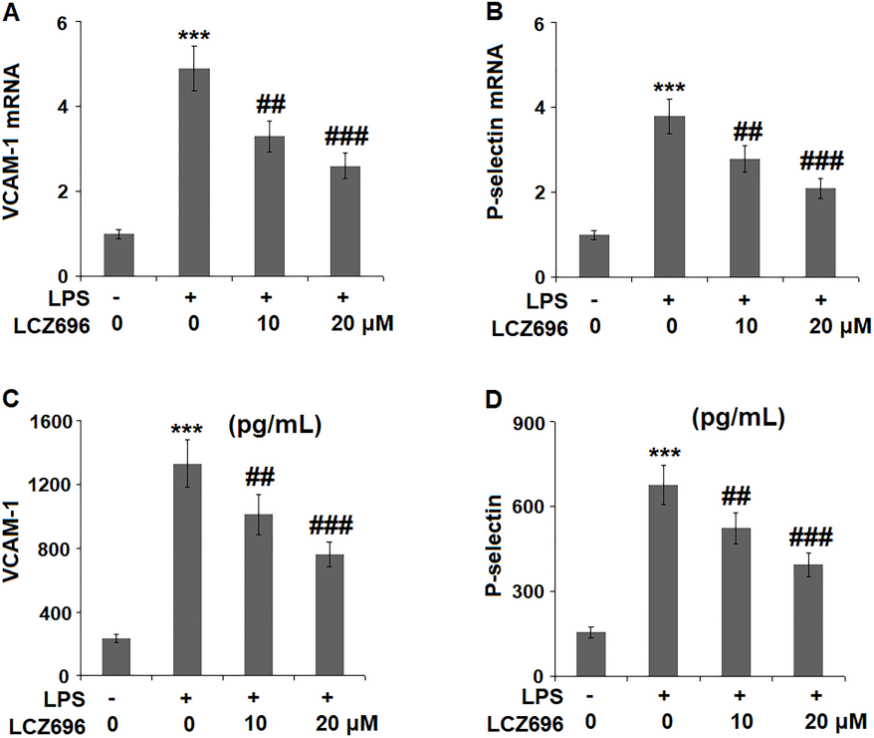
LCZ696 reduces LPS-induced expression of the cell adhesion molecules in HUVECs. Cells were stimulated with LPS (1 μg/mL) in the presence or absence of LCZ696 (10, 20 μM) for 24 hours. (**A**). mRNA of VCAM-1; (**B**). mRNA of P-selectin; (**C**). Protein of VCAM-1; (**D**). Protein of p-selectin (^***^P < 0.005 vs. control group; ^##, ###^*P* < 0.01, 0.005 vs. LPS group).

Endothelial cells expressed several cell adhesion molecules upon inflammatory stimuli, including VCAM-1 and P-selectin. At mRNA level, LPS alone induced 4.9 and 2.8-fold high of VCAM-1 and P-selectin. But a higher concentration of LCZ696 was able to reduce their transcription levels to only 2.6- and 2.1-fold, (Figure 6A, 6B) respectively. The results in Figure 6C and Figure 6D show that the protein levels of VCAM-1 and P-selectin were significantly increased from 235.6 and 156.5 pg/ml to 1331.8 and 678.1 pg/ml, respectively, by exposure to LPS alone. However, 10 and 20 μM LCZ696 reduced the protein levels of VCAM-1 to 1012.5 and 763.3 pg/mL, and P-selectin to 523.8 and 395.6 pg/mL, respectively.

### LCZ696 suppresses LPS-induced monocytes adhesion to endothelial cells

**Figure 7:**
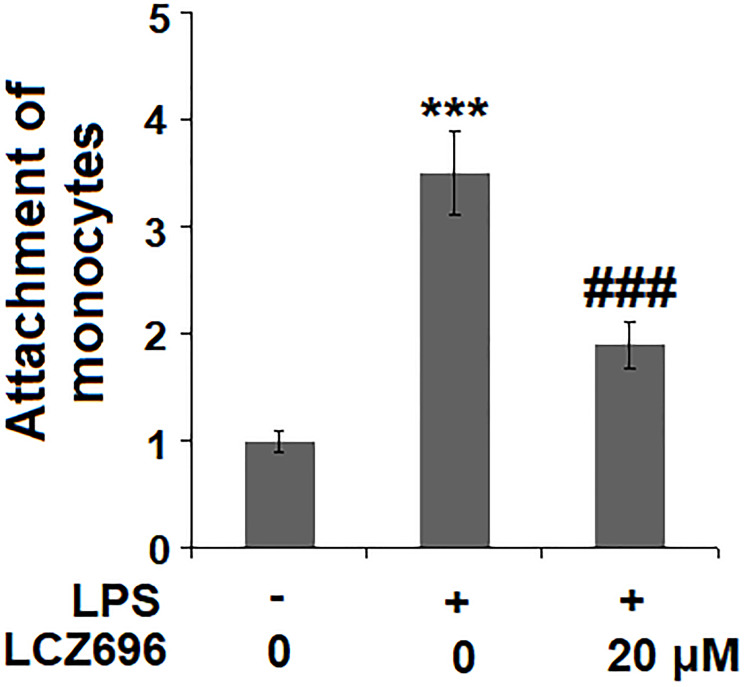
LCZ696 suppresses LPS-induced attachment of U937 monocytes to HUVECs. Cells were stimulated with LPS (1 μg/mL) in the presence or absence of LCZ696 (20 μM) for 24 hours. Attachment of U937 monocytes to HUVECs was measured by 5-chloromethylfluorescein diacetate (^***^P < 0.005 vs. control group; ^##, ###^*P* < 0.01, 0.005 vs. LPS group).

Based on the inhibitive effect of LCZ696 on inflammatory factors and endothelial adhesion molecules, its regulation on monocytes adhesion to endothelial cells was tested in a classic adhesion experiment. As expected, LPS alone promoted 3.5-fold more U937 monocytes adhesion to HUVECs but LCZ69 was able to suppress attached monocytes only to 1.9-fold, indicating a prominent suppression of monocytes adhesion to endothelial cells (Figure 7).

### LCZ696 suppresses the TLR4/Myd88 pathway

**Figure 8:**
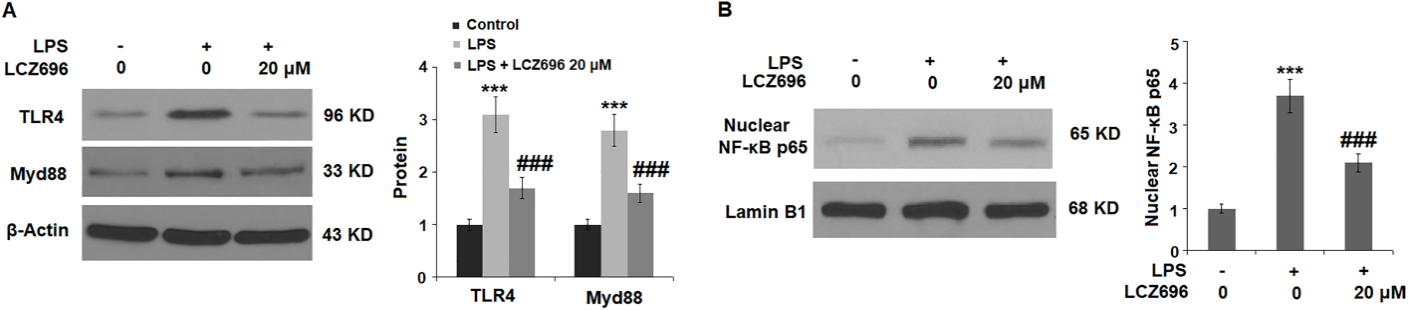
LCZ696 suppresses LPS-induced activation of the TLR4/Myd88 pathway in HUVECs. Cells were stimulated with LPS (1 μg/mL) in the presence or absence of LCZ696 (20 μM) for 24 hours. (**A**). The levels of TLR4 and Myd88 were measured using Western blot; (**B**). Nuclear levels of NF-κB p65 (^***^P < 0.005 vs. control group; ^##, ###^*P* < 0.01, 0.005 vs. LPS group).

Lastly, the potential molecular pathways that involved LCZ696 were explored. We focused on the TLR4/Myd88 sensor and nuclear NFκB p65 signals. As shown in Figure 8A, LPS alone induced 3.1- and 2.8-fold TLR4 and Myd88 expressions, but LCZ696 was able to reduce their expressions to 1.6- and 2.1-fold, respectively. As shown in Figure 8B, LPS alone induced increased nuclear expression of NFκB p65 to 3.7-fold, while LCZ696 reduced its expression to 2.1-fold only.

## DISCUSSION

Our study aims to examine the protective effects of the dual inhibition drug LCZ696 in cultured endothelial cells. LPS has been shown to induce endothelial cell death and alterations in the barrier function of the endothelium [15, 16]. By utilizing the LPS-induced HUVECs injury model, we show that LCZ696 has the protective capacity to suppress LPS-induced inflammation and ROS production in endothelial cells. Our findings reveal that LCZ696 inhibits all the tested pro-inflammatory cytokines and chemokines’ induction, including IL-1α, TNF-β, IL-6, CXCL1, and MCP-1. These mediators are critical factors in the inflammatory processes. Currently, endothelial cells have been recognized as an important source of multifunctional cytokine production, and endothelial-derived cytokines are known to be involved in hematopoiesis, immune response, coagulation, and other processes [17]. As a result, LCZ696 treatment significantly reduces LPS-induced intracellular ROS and MDA levels, which are lipid peroxidation products in endothelial cells [18].

One appealing fact is that our findings indicate that LCZ696 could suppress LPS-elicited VCAM-1 and P-selectin induction. At the cellular level, LCZ696 shows a potent suppression of LPS-caused monocytes adhesion to endothelial cells. Both VCAM-1 and P-selectin are induced on the atherosclerotic plaques and are involved in the recruitment of monocytes into sites of atherosclerosis [19, 20]. The expression of VCAM-1 is known to be closely associated with the atherosclerosis process [21]. The inhibitory effect of LCZ696 on the expression of vascular adhesion molecules and monocyte adhesion to endothelial cells suggests the drug could have a potential implication in suppressing vascular inflammation in the development of atherosclerosis.

Mechanistically, we explored the TLR4/Myd88 pathway as LPS is known to bind the TLR4 receptor in endothelial cells [5]. Endothelial TLR4 is the primary intravascular guarding system to detect bacterial invasion, and endothelial TLR4 is responsible for the recruitment of neutrophils to peripheral tissues in systemic endotoxemia [22]. When endothelial cells are exposed to LPS, the receptor complex comprising TLR4, CD14, and MD2 can recruit the adaptor protein MyD88 and activate the NF-κB pathway [6]. Our findings confirmed the inhibition by LCZ696 of the expressions of TLR4 and Myd88, and nuclear NF-κB p65 translocation, indicating that the inhibitory signal is transduced from the outside of a cell to the inside of the nucleus. In endothelial cells, NF-κB controls the global pro-inflammatory response such as the pro-atherogenic program [23]. Thus, we propose that the inhibitory effect of LCZ696 on pro-atherogenic IL-1α, TNF-β, IL-6, CXCL1, MCP-1, VCAM-1, and P-selectin is mediated by the TLR4/Myd88 and NF-κB pathways. The limitations of the study have to be mentioned. Firstly, the current study does not reveal how LCZ696 interferes with TLR4/Myd88 signals in endothelial cells. As the dual inhibitor for the AT1 receptor and neprilysin signaling, it remains unclear if the action of LCZ696 is dependent on these two pathways. The role of neprilysin in endothelial cells is not well known. One study found that neprilysin is expressed in endothelial cells, and its expression is regulated by physiological laminar shear [24]. Another study showed neprilysin is released from endothelial cells via exosomes [25]. The suppression of the endothelial AT1 pathway has been shown to improve endothelial function [12, 13], but the activation of the receptor stimulates the production of pro-inflammatory cytokines and enhances the activation of nuclear factor NF-κB [26]. A future mechanistic study is warranted to uncover if AT1 and neprilysin signals are involved in the modulation of LCZ696 in vascular endothelial cells. Secondly, we only investigated the beneficial effects of LCZ696 against LPS-induced damages in HUVECs. However, the pathological mechanisms of cardiovascular diseases are complex. A variety of risk factors have been reported to participate in the initiation and development of cardiovascular diseases, including genetics, aging, and obesity. Notably, in addition to endothelial cells, several other types of cells such as fibroblasts, vascular smooth muscle cells, and macrophages are reported to be involved in the pathophysiology of cardiovascular diseases. Animal models have been widely used for the study of pathological mechanisms and therapeutic strategy of cardiovascular diseases. Therefore, further *in vivo* studies with animal models or clinical trails will be helpful to verify the pharmacological function of LCZ696 in endothelial dysfunction and cardiovascular diseases.

In summary, this study reveals the protective effect of the dual inhibition drug LCZ696 in the context of endothelial injury by LPS. LCZ696 exhibits anti-inflammatory and anti-oxidative stress effects via the endothelial TLR4/Myd88 and NF-κB pathways. We conclude that LCZ696 could have potential implications in the modulation of vascular inflammation and atherosclerosis.

## MATERIALS AND METHODS

### Cell culture and treatment

Human umbilical vascular endothelial cells (HUVECs) were purchased from Lonza (CC-2635). The cells were grown in 2% serum endothelial growth media (EGM2) supplied with essential growth factors for no more than 7 passages. Human monocytes cells line U037 was from ATCC stock (TIB-202™) and grown in 10% fetal serum-containing DMEM media. All the cell culture was maintained in a 5% (v/v) CO_2_/ 95% (v/v) nitrogen incubator at 37°C. The cell culture grade, LPS, and LCZ636 were purchased from Sigma-Aldrich (St. Louis, USA). For LPS treatment, 1 μg/mL LPS containing growth media was added to confluent HUVECs media for 24 hours.10 and 20 μM LCZ636 and 1 μg/mL LPS were then added to confluent HUVECs media at the same time for 24 hours, for the LCZ636 co-treatment experiment. For the LCZ636 dose-responsive experiment, the cells were exposed to 0, 1, 5, 10, 20, 100, and 200 μM LCZ636 for 24 hours.

### MTT assay

The MTT assay was used to evaluate the effects of LCZ696 on cell viability. MTT is a cleaved product of mitochondrial dehydrogenase in the metabolically active cells and forms an insoluble formazan crystal. In brief, HUVECs were seeded at a density of 2 × 10^4^ cells/well in a 96-well plate. After treatment, 10 μl of MTT (5 μg/mL) (Sigma-Aldrich, USA) were added to each well in the plate and incubated for 4 hours at 37°C. Then, 100 μl of DMSO reagent were added to the well and incubated at 37°C. The absorbance was measured at 570 nm using a multi-well spectrophotometer (BioTek, USA).

### DCFH-DA staining

The cellular ROS were measured using a cell-permeable fluorescent probe, 2′7′-dichlorofluorescin diacetate (DCFH-DA, D6665; Sigma-Aldrich, St. Louis, MO) method, based on the ROS-dependent oxidation of DCFH-DA to DCF. Briefly, HUVECs plated on cover slides in 6-well plates were grown to 50–80% confluence. The cells were treated with LPS in the presence or absence of LCZ696. The cells were washed using PBS and 200 μl DCFH-DA (10 μM) was added for 30 minutes at 37°C in the dark. Upon intracellular oxidation, DCFH-DA turned to fluorescent DCF was measured using an inverted Olympus microscope (Olympus, Tokyo, Japan). Three replicate plates were measured for each group sample, and the Mean fluorescent intensity of DCF was calculated with Image-Pro Plus (version 5.0, Media Cybernetics, USA.) to obtain the intracellular ROS levels.

### MDA assay

To assess cellular lipid peroxidation, the activity of lipid peroxidation (MDA) in HUVECs was measured. In brief, the treated HUVECs were lysed with RIPA buffer and centrifuged at 13,500 × g for 5 minutes at 4°C, the supernatants were collected and the protein concentrations in the supernatants were measured using a BCA protein assay kit (Thermo Fisher Scientific, USA). The MDA activity was measured using a commercial MDA assay kit (ABCAM, cat# ab118970). The measuring procedures were performed following the corresponding manufacturers' protocols.

### Real-time PCR analysis

Total RNAs from cultured HUVECs in 6-well plates were extracted using an RNeasy Mini Kit (Qiagen, Shanghai). The concentrations and quality of RNAs were determined using a Nanodrop 2000 spectrophotometer (Thermo Fisher Scientific, Waltham, U.S.A.). 1 μg total RNA from the sample was reverse transcribed using a One Step PrimeScript RT-PCR Kit (Takara Ltd., Japan). A Real-time PCR reaction was performed on a CFX Connect Real-Time PCR Detection System (BioRad, USA). The fast PCR master mix (Roche, Shanghai) with the primers mixture was reacted in the following steps: pre-denaturation at 94°C for 3 minutes, 40 cycles of denaturation at 94°C for 20 seconds, annealing at 60°C for 30 seconds, and extension at 68°C for 10 seconds. GAPDH was served as a housekeeping control, and a 2^-ΔΔCT^ formula was used to calculate the relative expression levels of the target genes. The primers used in this study are listed in Table 1.

**Table 1:** The primers sequences.

**Target gene**	**Upstream Sequence (5′-3′)**	**Downstream Sequence (5′-3′)**
**IL-6**	AGGGCTCTTCGGGAAATGTA	TGCCCAGTGGACAGGTTTC
**TNF-β**	GCTGCTCACCTCATTGGAGAC	CACCATCTTCTGGGAGCTGAG
**MCP-1**	TTCTGTGCCTGCTGCTCAT	GGGGCATTGATTTGCATCT
**CXCL1**	TCTC ATCCAGAGCTTGAAGGTGTTG	GTCTGTCTTCTTTCTCCGTTACTT
**VCAM-1**	CGG- CTTAAAATGCCTGGGAAGATGGT	GTCAATGAGACGGAGTCACCAAT
**P-selectin**	GAAC CTATACCTGCTCCTGCTACCCA	CTGGAGTCGTAGGCAAAGGC
**IL-1α**	AGCCCATGATTTAGAGACCAT	TGATGAACTCCTGCTTGACGAT
**GAPDH**	CCTCGTCCCGTAGACAAAATG	TGAGGTCAATGAAGGGGTCGT

### ELISA

The supernatants of HUVECs were collected and centrifuged at 14,000 rpm/10 minutes to remove any cell debris. The soluble portion of protein samples was quantified using a BCA protein assay kit as described previously. The ELISA kit was taken out from the 4°C, refrigerator and maintained at room temperature for 30 minutes. The ELISA kits for IL-6 (Cat#PD6050), TNF-β (Cat#DY211), IL-1α (Cat#SLA50), CXCL1 (Cat#SGR00B), MCP-1 (Cat#PDCP00), VCAM-1 (Cat#DVC00), and P-Selectin (Cat#DPSE00) were purchased from R&D (Minneapolis, USA), and the assay experiments were performed according to the protocols. The absorbance values were measured using a microplate reader (BioTek, USA) at 450 nm.

### Isolation of Nuclear fragmentation and Western blot analysis

The nuclear protein from HUVECs was extracted using a commercial nuclear and cytoplasmic extraction kit (Thermo Scientific Inc. USA). The isolation procedures followed were according to the manufacturer’s instruction. Whole cell lysates were lysed with RIPA buffer (20 mM Tris-HCl at pH 7.5, 150 mM NaCl, 1 mM Na_2_EDTA, 1 mM EGTA, 1% NP-40, and 1% sodium deoxycholate) supplied with protease inhibitor (Roche, Shanghai). The protein samples were quantified using a BCA protein assay kit (Thermo Fisher Scientific, USA). 10–20 μg of protein were separated on an 8–16% SDS-PAGE gel and transferred to a nitrocellulose membrane. The membranes were then blocked with 5% BSA-PBST and incubated with the primary antibodies overnight at 4°C. The primary antibodies include: TLR4 (1:1000, #14358, Cell Signaling Technologies, USA), Myd88 (1:2000, #50010, Cell Signaling Technologies, USA), NF-κB p65 (1:2000, #8242, Cell Signaling Technologies, USA), Lamin B1 (1:3000, #MAB8525, R&D systems, USA) and β-actin (1:10000, #MAB8929, R&D systems, USA). Following extensive washing, the blots were incubated with appropriate HRP-conjugated secondary antibodies (1: 10,000 dilution) at room temperature for 1 hour, and visualized using the Amersham ECL method (GE, Shanghai). Images were visualized and quantified with Gel Doc software (Bio-Rad Laboratories, USA). The digital values were normalized to the sample loading control. The results were expressed as a fold change relative to the control group.

### CMFDA labeling and monocytes attachment

U937 monocytes were labeled with 15 μM 5-chloromethylfluorescein diacetate (CMFDA) (Life Technologies) for 45 minutes at 37°C followed by washing twice with the medium. For endothelial cell-monocyte adhesion, HUVECs were stimulated with 1 μg/mL LPS in the presence or absence of 10 and 20 μM LCZ636 and incubated with CMFDA-labeled U937 monocytes for 30 minutes at 37°C, followed by washing twice with warm PBS. The number of adherent U937 cells per visible field was determined by recording a video using the Leica DMS300 digital microscope (Leica, Shanghai), Images were recorded and the attached cells were quantified from 10 frames in the off-line analysis using Metamorph automation software (Molecular Devices, USA).

### Statistical analysis

The data are expressed as Mean ± standard deviation (SD). Statistical significance between groups was determined using the ANOVA test followed by a posthoc Tukey’s HSD test. The differences between groups were determined as significant with *p* < 0.05 as the threshold. Statistical analysis was performed using the GraphPad Prism 5 software (GraphPad Prism USA).
